# Inhibition of NMDA Receptors Prevents the Loss of BDNF Function Induced by Amyloid β

**DOI:** 10.3389/fphar.2018.00237

**Published:** 2018-04-11

**Authors:** Sara R. Tanqueiro, Rita M. Ramalho, Tiago M. Rodrigues, Luísa V. Lopes, Ana M. Sebastião, Maria J. Diógenes

**Affiliations:** ^1^Instituto de Farmacologia e Neurociências, Faculdade de Medicina, Universidade de Lisboa, Lisbon, Portugal; ^2^Instituto de Medicina Molecular, Faculdade de Medicina, Universidade de Lisboa, Lisbon, Portugal; ^3^Department of Ophthalmology, Centro Hospitalar e Universitário de Coimbra, Coimbra, Portugal

**Keywords:** Alzheimer’s disease, brain-derived neurotrophic factor, TrkB receptor, extrasynaptic N-methyl-d-aspartate receptors, memantine, long-term potentiation, spine density, synaptic plasticity

## Abstract

Brain-derived neurotrophic factor (BDNF) plays important functions in cell survival and differentiation, neuronal outgrowth and plasticity. In Alzheimer’s disease (AD), BDNF signaling is known to be impaired, partially because amyloid β (Aβ) induces truncation of BDNF main receptor, TrkB-full length (TrkB-FL). We have previously shown that such truncation is mediated by calpains, results in the formation of an intracellular domain (ICD) fragment and causes BDNF loss of function. Since calpains are Ca^2+^-dependent proteases, we hypothesized that excessive intracellular Ca^2+^ build-up could be due to dysfunctional N-methyl-d-aspartate receptors (NMDARs) activation. To experimentally address this hypothesis, we investigated whether TrkB-FL truncation by calpains and consequent BDNF loss of function could be prevented by NMDAR blockade. We herein demonstrate that a NMDAR antagonist, memantine, prevented excessive calpain activation and TrkB-FL truncation induced by Aβ_25–35_. When calpains were inhibited by calpastatin, BDNF was able to increase the dendritic spine density of neurons exposed to Aβ_25135_. Moreover, NMDAR inhibition by memantine also prevented Aβ-driven deleterious impact of BDNF loss of function on structural (spine density) and functional outcomes (synaptic potentiation). Collectively, these findings support NMDAR/Ca^2+^/calpains mechanistic involvement in Aβ-triggered BDNF signaling disruption.

## Introduction

Brain-derived neurotrophic factor (BDNF) is a neurotrophin widely expressed in the central nervous system that, through activation of its full-length receptor (TrkB-FL), plays pivotal roles in cell survival and differentiation, axon elongation, dendritic growth and synaptic plasticity (Lewin and Barde, 1996; Lu et al., 2013). Truncated isoforms of TrkB receptors (TrkB-TC) act as negative modulators of TrkB-FL receptors (Eide et al., 1996; Dorsey et al., 2006), and changes in TrkB-FL:TrkB-TC ratio are thought to cause and/or reflect BDNF signaling dysfunction (Gomes et al., 2012). Cultured rat hippocampal or striatal neurons under excitotoxic conditions, such as exposure to glutamate, present a downregulation of TrkB-FL and upregulation of TrkB-TC receptors (Gomes et al., 2012), which may result in dysregulated actions of BDNF. Excitotoxicity and dysregulation of BDNF signaling are involved in several pathological processes, such as brain ischemia (Ferrer et al., 2001; Vidaurre et al., 2012; Tejeda et al., 2016), traumatic brain injury (Schober et al., 2012; Rostami et al., 2014) and neurodegenerative diseases (Porritt et al., 2005; Plotkin et al., 2014; Nguyen et al., 2016), including AD (Jerónimo-Santos et al., 2015). The link between excitotoxicity in AD and BDNF dysregulation is however not fully understood.

In AD, total BDNF levels are decreased and the TrkB-TC:TrkB-FL ratio is increased in hippocampal and cortical *post-mortem* samples from AD patients (Phillips et al., 1991; Connor et al., 1997; Allen et al., 1999; Ferrer et al., 1999; Holsinger et al., 2000). Such imbalance was suggested to contribute to cognitive impairment in AD (Morris, 1993). On the other hand, boosting BDNF/TrkB signaling was shown to ameliorate synaptic function and cognitive decline in mouse models of AD (Blurton-Jones et al., 2009; Nagahara et al., 2009; Devi and Ohno, 2012; Kemppainen et al., 2012).

We have previously demonstrated that Amyloid-β peptide (Aβ) – one of the main molecular drivers of the disease (Ittner and Götz, 2011) – is able to increase the expression of truncated isoforms of TrkB receptor in primary neuronal cultures (Kemppainen et al., 2012). Moreover, we showed that Aβ induces a calpain-dependent cleavage of TrkB-FL, which results in the formation of a previously unidentified truncated isoform (TrkB-T’) and of an ICD fragment (Jerónimo-Santos et al., 2015).

Calpains are intracellular Ca^2+^-dependent proteases that play physiological roles (Goll et al., 2003; Ono and Sorimachi, 2012). However, calpains were also reported to be dysregulated in aging-related diseases, such as AD (Nixon, 2003; Vosler et al., 2008), leading to excitotoxic neuronal death (Caba et al., 2002), synaptic dysfunction and spatial memory impairments (Trinchese et al., 2008; Granic et al., 2010; Medeiros et al., 2012). Excessive activation of calpains might result from increased intracellular Ca^2+^ concentrations that occur in excitotoxic conditions (Kelly and Ferreira, 2006, 2007). One source of intracellular Ca^2+^ are NMDARs, which have been implicated in excitotoxicity phenomena (Rueda et al., 2016). In animal models of AD, Aβ accumulation may lead to abnormal NMDAR activation, even in early stages (Parameshwaran et al., 2008). Moreover, Aβ was shown to induce a sustained Ca^2+^ influx by directly interacting with NMDARs, especially with those found at extrasynaptic sites (eNMDARs) (Alberdi et al., 2010; Texidó et al., 2011; Ferreira et al., 2012).

Thus, we hypothesized that eNMDARs could act as mediators of Aβ toxicity, by promoting Ca^2+^ influx and calpain activation, which would then lead to TrkB-FL truncation and BDNF signaling disruption. In order to experimentally address this hypothesis, we used the only NMDAR antagonist commercially available for the treatment of AD – memantine – as a pharmacological tool to preferentially block eNMDARs (Lipton, 2007; Parsons et al., 2007; Xia et al., 2010). Memantine, at doses that translate into plasmatic concentrations that are known to be highly selective to eNMDAR, has been shown to benefit cognitive function, a global outcome in patients with moderate to severe AD (Lipton, 2007; Parsons et al., 2007; Xia et al., 2010). Briefly, we herein show that memantine was able to reduce Aβ-induced TrkB-FL cleavage and to restore BDNF-mediated actions on spine density. In this regard, spine density was reported to be reduced in brain samples of both AD patients (Knobloch and Mansuy, 2008) and animal models (Spires et al., 2005; Spires-Jones et al., 2007), which has been related to the cognitive deficits. Importantly, BDNF is known to increase the number of dendritic spines (Tyler and Pozzo-Miller, 2001; Ji et al., 2005, 2010; Kellner et al., 2014), whereby promoting synaptic strength. Moreover, we show that memantine prevented Aβ-induced loss of BDNF effect on LTP, which is accepted as the synaptic correlate of learning and memory (Bliss and Collingridge, 1993). Collectively, our data support the thesis that NMDAR dysregulation may be mechanistically implicated in Aβ-induced BDNF signaling impairment.

## Materials and Methods

### Animals and Brain Areas Used

Sprague-Dawley and Wistar rats (Charles River, Barcelona, Spain) were maintained in controlled temperature (21 ± 1°C) and humidity (55 ± 10%) conditions with a 12:12 h light/dark cycle and access to food and water ad libitum. All animals were handled according to Portuguese Law and the European Union Directive (86/609/EEC) on the protection of animals for scientific experimentation. Care was taken to minimize the number of animals sacrificed.

Rats were deeply anesthetized with isoflurane (Esteve, Barcelona, Spain) and sacrificed for tissue preparation. The hippocampus was used for functional studies for two reasons: it is one of the brain areas more affected in AD and where BDNF effects have been most extensively studied (Figurov et al., 1996; Korte et al., 1998; Pang and Lu, 2004). On the other hand, since Aβ-induced dysregulation of TrkB receptors was reported to be similar in cortical and hippocampal cultures (Kemppainen et al., 2012), cortical cultures were used for molecular studies in order to increase culture yield and reduce the number of animals used.

### Amyloid-β Peptides

All the experiments were performed using Aβ_25–35_ (Bachem, Bubendorf, Switzerland). As previously confirmed, Aβ_25-35_, which contains mainly protofibrillar and fibrillar amyloid structures (Del Mar Martínez-Senac et al., 1999; Kemppainen et al., 2012), represents the biologically active region of Aβ and induces the same molecular and cellular dysfunction as Aβ_1–42_, similar to what has been observed in AD brains (Pike et al., 1995; Kaminsky et al., 2010). Stock solutions of Aβ_25–35_ were prepared in MilliQ water to a final concentration of 1 mg/mL. We used 25 μM of Aβ_25–35_, as previously (Kemppainen et al., 2012; Jerónimo-Santos et al., 2015).

### Primary Neuronal Cultures and Drug Treatments

Primary neuronal cultures were obtained from fetuses of 18/19-day pregnant Sprague-Dawley females. Unless stated otherwise, culture reagents and supplements were purchased from Gibco (Paisley, United Kingdom). The fetuses were collected in HBSS and, after brain dissection, the cerebral cortex was isolated, and the meninges were removed. The tissue was mechanically fragmented, and its digestion was performed with 0.025% (wt/vol) of trypsin solution in HBSS for 15 min at 37°C. After tissue digestion, cells were precipitated by centrifugation at 1200 rpm. The supernatant was removed and 20% of Fetal Bovine Serum was added to HBSS. Cells were again precipitated by centrifugation, the supernatant removed and 2 mL of HBSS was added to the solution. Cells resuspension by pipette aspiration was required between centrifugations in order to dissociate cells. This washing process was repeated four more times to neutralize trypsin. After washed, cells were resuspended in supplemented Neurobasal medium (0.5 mM L-glutamine, 25 mM glutamic acid, 2% B-27, and 25 U/mL penicillin/streptomycin). To obtain single cells and avoid cellular clusters or tissue fragments, the suspension was filtrated with a nylon filter (BD Falcon^TM^ Cell Strainer 70 μM, Thermo Fisher Scientific, Waltham, MA, United States). Cells were plated at densities of 6 × 10^4^ and 5 × 10^4^ cells/cm^2^ on coverslips for western blotting and immunocytochemistry experiments, respectively, and maintained at 37°C in a humidified atmosphere of 5% CO_2_. These coverslips were previously sterilized under UV light, coated overnight with 10 μg/mL of poly-D-lysine (Sigma-Aldrich, St. Louis, MO, United States) and then washed with sterile H_2_O. Primary neuronal cultures were incubated with 25 μM of Aβ_25–35_ at DIV13 for 24 h at 37°C, as previously described (Kemppainen et al., 2012; Jerónimo-Santos et al., 2015). In these experiments, cells were also co-incubated with Aβ_25–35_ and 1 μM memantine, a NMDAR antagonist (Sigma-Aldrich). Finally, for immunocytochemistry, cells were co-incubated with the same drugs and 1 μM of calpastatin (Millipore, Billerica, MA, United States), a cell-permeable calpain inhibitor, in the presence or absence of 20 ng/mL of BDNF, a gift from Regeneron Pharmaceuticals (Tarrytown, NY, United States). BDNF was used at a final concentration of 20 ng/mL (corresponding to ∼0.75 nM).

### Western Blot (WB)

After treatments, primary neuronal cultures at DIV14 were washed with ice-cold PBS and lysed with Radio Immuno Precipitation Assay buffer (RIPA) [50 mM Tris–HCl (pH 7.5), 150 mM NaCl, 5 mM ethylenediaminetetraacetic acid, 0.1% SDS and 1% Triton X-100] containing protease inhibitors (Roche, Penzberg, Germany). Adherent cells were scraped off the dish using a cell scraper and the cell suspension were centrifuged at 13,000 *g*, 4°C during 10 min. The supernatant was aspired, discarding the pellet, and placed in fresh tubes. The amount of protein was determined by Bio-Rad DC reagent (Bio-Rad Laboratories, Berkeley, CA, United States) and all samples were prepared with the same amount of total protein (30 μg). A loading buffer (350 mM Tris pH = 6.8, 10% SDS, 30% glycerol, 600 mM Dithiothreitol, 0.06% bromophenol blue) was added and the mixture was boiled at 95–100°C for 5 min. Next, all samples and the molecular weight marker (Thermo Fisher Scientific) were loaded and separated on 10% SDS–polyacrylamide gel electrophoresis (SDS–PAGE) within a standard migration buffer (25 mM Tris pH = 8.3, 192 mM Glycine, 10% SDS), at a constant voltage between 80 and 120 mV. Then, proteins were transferred onto PVDF membranes (GE Healthcare, Buckinghamshire, United Kingdom), previously soaked in methanol for 5 min, within the standard buffer (25 mM Tris pH = 8.3, 192 mM Glycine, 15% methanol) for wet transfer conditions. After 1.5 h of transfer, membranes were soaked again in methanol for 5 min and then stained with Ponceau S solution to evaluate protein transference efficacy. Membranes were blocked with a 5% (w/v) non-fat dry milk solution in Tris-buffered saline with the detergent Tween-20 (20 mM Tris base, 137 mM NaCl and 0.1% Tween-20). Membranes were incubated overnight with primary antibodies: C-14 – the C-terminal of TrkB-FL rabbit polyclonal antibody (1:2000) and the αII-Spectrin (C-3) mouse monoclonal antibody (1:2500), raised against human spectrin (aa. 2368–2472) (Santa Cruz Biotechnology, Dallas, TX, United States) and 1 h at RT with goat anti-mouse and goat anti-rabbit IgG-horseradish peroxidase-conjugated secondary antibodies (Santa Cruz Biotechnology). Immunoreactivity was visualized using ECL chemiluminescence detection system (GE Healthcare), band intensity was measured using ChemiDoc (Bio-Rad Laboratories) and quantified by the digital densitometry ImageJ 1.45 software (Bethesda Softworks, Bethesda, MD, United States). The intensity of GAPDH was used as loading control.

### Immunocytochemistry

Primary neuronal cultures at DIV14 were washed with PBS and then fixed in 4% paraformaldehyde in PBS (pH = 7.4) for 15 min at RT. Cells were incubated with the blocking solution (3% (w/v) bovine serum albumin) (Sigma-Aldrich) in PBS with 0.1% (v/v) Triton X-100 for 1 h. After new washes with PBS, cells were incubated with mouse microtubule-associated protein 2 (MAP2) primary antibody (1:200 in blocking solution) (Millipore), to specifically detect neurons, overnight at 4°C. After this, cells were washed with PBS and then incubated with Goat Anti-Mouse Alexa Fluor^®^ 568 secondary antibody (1:200 in blocking solution) (Invitrogen), for 1 h at RT, in the dark. The secondary antibody solution was decanted and rinsed with PBS. Then, cells were incubated with Alexa Fluor^®^ 488 Phalloidin (1:40 in PBS) (Invitrogen), which recognizes filamentous actin *(F-actin)*, for 30 min. *F-actin* has an important role in the constitution of the cytoskeleton of dendritic spines (Koskinen and Hotulainen, 2014). After being washed, coverslips were mounted in Mowiol mounting solution and observed using an inverted fluorescent microscope Axiovert 135 TV (Carl Zeiss Microscopy, Thornwood, NY, United States). Spine density was counted as the number of protrusions per 10 μm of the parent dendrite, as previously reported (Alonso et al., 2004; Ji et al., 2010) with a distance of 25 μm from the cell body. We analyze 6 neurons per condition and, for each neuron, we counted protrusions in 3 different dendrites.

### Freshly Prepared Hippocampal Slices

For electrophysiological studies, male Wistar rats (8–12 weeks old) were sacrificed after being deeply anesthetized with isoflurane. The brain was quickly removed and placed in ice-cold continuously oxygenated (O_2_/CO_2_: 95%/5%) aCSF (124 mM NaCl, 3 mM KCl, 1.2 mM NaH_2_PO_4_, 25 mM NaHCO_3_, 2 mM CaCl_2_, 1 mM MgSO_4_, and 10 mM glucose, pH 7,4) and the hippocampi were dissected free. The hippocampal slices were cut perpendicularly to the long axis of the hippocampus with a McIlwain tissue chopper (400 μm thick). After recovering functionally and energetically for at least 1 h in a resting chamber filled with oxygenated aCSF at RT, slices were incubated for 3 h either with aCSF (control), Aβ_25–35_ peptide (25 μM) alone, memantine (1 μM) alone or with both Aβ_25–35_ and memantine.

### *Ex Vivo* Electrophysiology Recordings

Hippocampal slices were transferred to a recording chamber continuously superfused with oxygenated aCSF at 32°C (flow rate of 3 mL/min). fEPSPs were recorded extracellularly through a microelectrode filled with 4 M NaCl (2–6 MΩ) placed in the stratum radiatum of the CA1 area. Two different pathways of Schaffer collateral fibers were stimulated (rectangular pulses of 0.1 ms duration) at every 10 s by two bipolar concentric wire electrodes place on the Schaffer fibers. Recordings were obtained with an Axoclamp 2B amplifier (Axon Instruments, Foster City, CA, United States), digitized and continuously stored on a personal computer with the LTP software (Anderson and Collingridge, 2001). Individual responses were monitored and averages of six consecutive responses were obtained and the slope of the initial phase of the fEPSP was quantified. LTP induction and quantification were performed as described previously (Diogenes et al., 2011). Since the facilitatory action of BDNF upon LTP is mostly seen under weak 𝜃-burst protocol (three trains of 100 Hz, three stimuli, separated by 200) (Fontinha et al., 2008), LTP was induced by this protocol. Moreover, this pattern of stimulation is considered to be closer to what occurs in the hippocampus during episodes of learning and memory in living animals (Albensi et al., 2007). Therefore, we selected the more adequate stimulation paradigm to detect BDNF effect upon LTP (Fontinha et al., 2008), so that we could evaluate the modulatory influence of Aβ and memantine. After a stable fEPSP slope LTP was inducted in the first pathway. After 60 min of LTP induction, BDNF (20 ng/mL) was added to the superfusion solution. The intensity of stimulation was adjusted for similar values recorded before BDNF application. After at least 20 min of BDNF perfusion, LTP was induced in the second pathway. BDNF remained in the bath until the end of the experiment. LTP was quantified as % change in the average slope of the fEPSP taken from 46 to 60 min after LTP induction relatively to the average slope of the fEPSP measured during the 10 min before LTP induction. BDNF effect on LTP was evaluated by comparing the LTP magnitude in the first (control pathway, aCSF superfusion) and second pathway (test pathway, BDNF superfusion).

### Statistical Analysis

Data are expressed as mean ± standard error of the mean (SEM) of the *n* number of independent experiments. Independent experiments are considered the results observed in different primary neuronal cultures obtained from fetuses of different pregnant-Sprague Dawley females and the results acquired from hippocampal slices of different Wistar rats.

**FIGURE 1:**
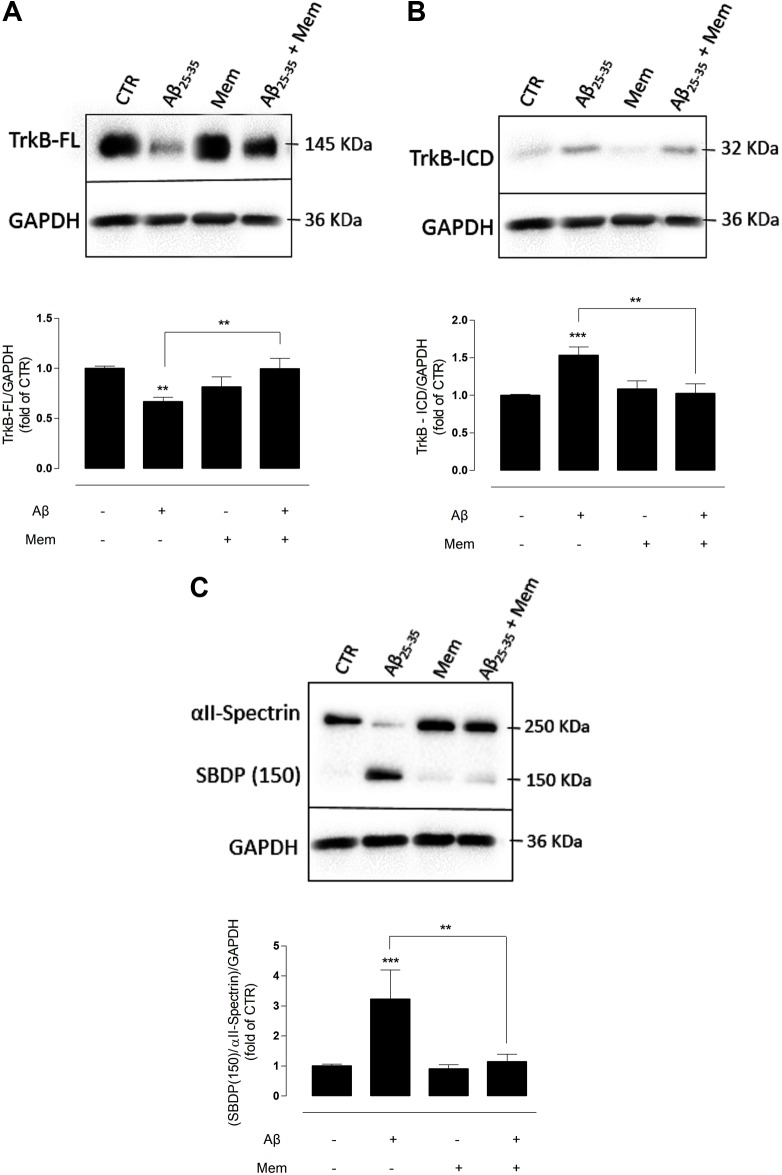
The inhibition of eNMDAR reduces the cleavage of TrkB-FL by modulating calpains activation. **(A)** TrkB-FL protein levels. Representative western-blots of DIV14 neuronal cultures showing the effect of 24 h of 25 μM Aβ_25–35_ and 1 μM memantine on TrkB-FL (∼145 kDa). The panel shows the average band intensity of TrkB-FL (^∗∗^*p* < 0.01, *n* = 9–22, two-way ANOVA with Tukey *post hoc* test). **(B)** TrkB-ICD protein levels. Representative western-blots of DIV14 neuronal cultures showing the effect of 24 h of 25 μM Aβ_25–35_ and 1 μM memantine on TrkB-ICD (∼32 kDa). The panel shows the average band intensity of TrkB-ICD (^∗∗^*p* < 0.01, ^∗∗∗^*p* < 0.001, *n* = 10–20, two-way ANOVA with Tukey *post hoc* test) and **(C)** Calpains activation. Representative western-blots of DIV14 neuronal cultures showing the effect of 24 h of 25 μM Aβ_25–35_ and 1 μM memantine on SBDP (150 kDa). The panel shows the average ratio of the SBDP (150) to intact spectrin (^∗∗^*p* < 0.01, ^∗∗∗^*p* < 0.001, *n* = 7–20, two-way ANOVA with Tukey *post hoc* test). Results were normalized to the loading control, GAPDH. Values are mean ± SEM.

**FIGURE 2:**
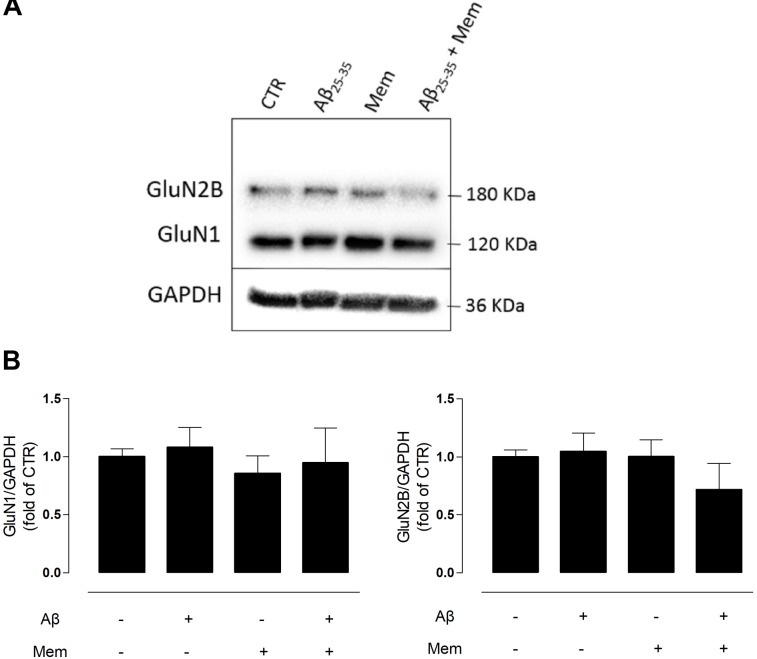
Neither Aβ nor memantine caused significant changes in different NMDAR subunits expression. **(A)** Representative western-blots of DIV14 neuronal cultures showing the effect of 24 h of 25 μM Aβ_25–35_ and 1 μM memantine on GluN1 and GluN2B. **(B)** The panels show the average band intensity of GluN1 (left histogram) and GluN2B (right histogram). Results were normalized to the loading control, GAPDH. Values are mean ± SEM.

**FIGURE 3:**
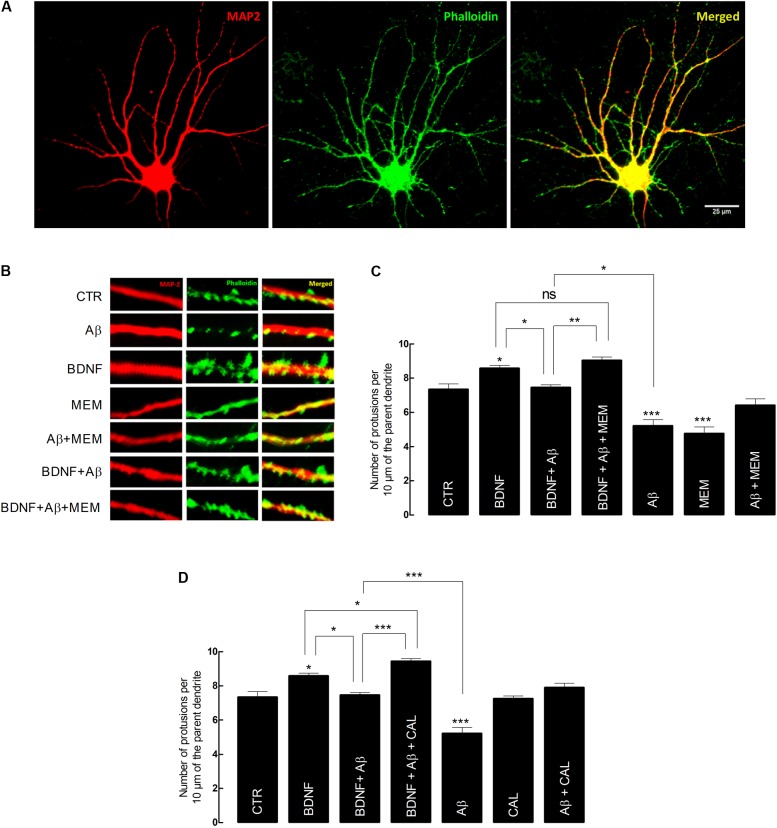
Brain-derived neurotrophic factor (BDNF) restores its capacity to increase spine density after inhibition of Aβ-induced NMDAR activation. **(A)** Representative image of an untreated neuron obtained from primary cultures. DIV14 neurons were incubated with BDNF (20 ng/mL) for 24 h, in the presence or absence of Aβ_25–35_ (25 μM) and/or memantine (1 μM) or calpastatin (1 μM). MAP2 (red) specifically detects neurons, while phalloidin (green), recognizes F-actin, thus labeling protrusions (filopodia and spines). The merge of both elements is represented in yellow. Six neurons were analyzed per condition and spine density was considered, in each cell, as the number of protrusions per 10 μm of the parent dendrite with a distance of 25 μm from the cell body. Protrusions were counted in each neuron in three different dendrites. **(B)** Treatments effects on synaptic growth. Aβ significantly reduces the number of protrusions, whereas BDNF increases the number of protrusions when incubated alone. In the presence of Aβ, BDNF loses its ability to increase the number of protrusions, which is rescued when cells are incubated with memantine. **(C)** The panels show the average number of protrusions in different conditions when neurons were treated with memantine and **(D)** calpastatin (^∗^*p* < 0.05, ^∗∗^*p* < 0.01, ^∗∗∗^*p* < 0.001, *n* = 6–14, three-way ANOVA with Tukey *post hoc* test). Values are mean ± SEM.

**FIGURE 4:**
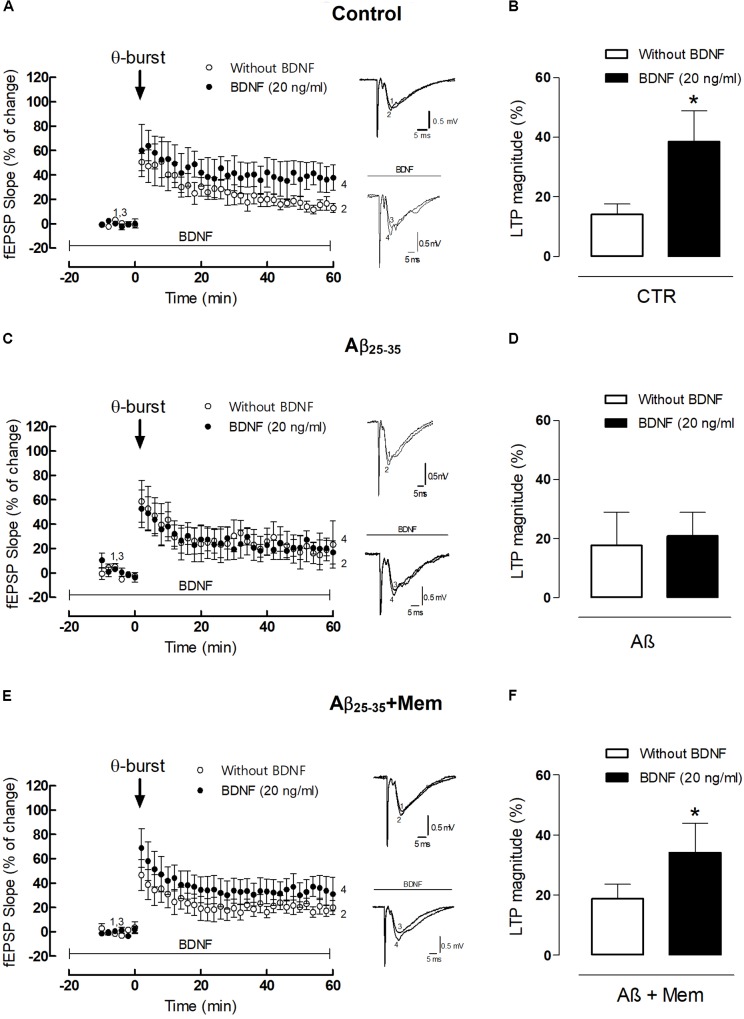
The inhibition of NMDAR activation by Aβ restores the facilitatory effect of BDNF upon 𝜃-burst-induced LTP. **(A,C,E)** Panels in the left side show the averaged time courses changes in field excitatory post-synaptic potential (fEPSP) slope induced by a 𝜃-burst stimulation in the absence or in the presence of BDNF (20 ng/mL) in the second stimulation pathway in rat hippocampal slices without (**A**, *n* = 4) or with a pre-exposure for 3 h to aCSF solution containing 25 μM Aβ_25–35_ (**C**, *n* = 3) and 25 μM Aβ_25–35_ in the presence of 1 μM Mem (**E**, *n* = 7). Tracings from representative experiments are shown to the right of panels **A,C,E**; each tracing is the average of six consecutive responses obtained before (1 and 3) and 46–60 min after (2 and 4) LTP induction. Tracings are composed by the stimulus artifact, followed by the pre-synaptic volley and the fEPSP. Tracings 1 and 2 and tracings 3 and 4 were obtained in the absence and presence of BDNF, respectively. **(B,D,F)** Histograms depicting LTP magnitude (change in fEPSP slope at 46–60 min) induced by 𝜃-burst stimulation in the presence and absence of BDNF for each group of pretreated slices (control, Aβ_25–35_, Aβ_25–35_+Mem). (^∗^*p* < 0.05, *n* = 3–7, paired *t*-test) Values are mean ± SEM.

For the analysis of the experiments reported in **Figures 1**, **2** and a two-way ANOVA model was used, considering two fixed factors (exposure to Aβ_25–35_ and memantine), each with two levels (presence versus absence). For the experiments in **Figure 3**, three-way ANOVA models were built, where three fixed factors (exposure to Aβ_25–35_, memantine/calpastatin and BDNF) were considered, each with two levels (presence versus absence). The mean squares, *F*-values and *p*-values of each source of variance within all ANOVA models built is provided as Supplementary Tables 1–7. Whenever a significant interaction was detected, a Tukey *post hoc* test was performed for multiple pairwise comparisons. For the experiments in **Figure 4**, paired *t*-tests were used to compare LTP magnitudes in the control and test pathways of the same slice.

All statistics were performed on STATA (version 14.2, StataCorp LCC, College Station, TX, United States). *P* < 0.05 were considered statistically significant. Graphical representations were built on GraphPad (version 6.0f, La Jolla, CA, United States).

## Results

### The Inhibition of NMDAR Limits TrkB-FL Cleavage Induced by Aβ

Primary cultured cortical neurons were treated at DIV13 with Aβ_25–35_ fragment (25 μM) for 24 h, either in the presence or absence of memantine at a concentration (1 μM) highly selective to eNMDARs (Lipton, 2007; Parsons et al., 2007; Xia et al., 2010). Total proteins were then isolated, and TrkB-FL and TrkB-ICD levels were evaluated by WB.

Exposure to Aβ_25–35_ alone induced a marked decrease in TrkB-FL receptor levels (TrkB-FL_Aβ_: 0.67 ± 0.06; TrkB-FL_CTR_: 1.00 ± 0.04, *n* = 10–22, *p* = 0.001, two-way ANOVA with Tukey *post hoc* test, **Figure 1A** and Supplementary Table 1) and a reciprocal increase in TrkB-ICD levels (TrkB-ICD_Aβ_: 1.53 ± 0.10; TrkB-ICD_CTR_: 1 ± 0.07, *n* = 10–20, *p* < 0.001, two-way ANOVA with Tukey *post hoc* test, **Figure 1B** and Supplementary Table 2), as previously described (Jerónimo-Santos et al., 2015). Importantly, co-incubation with memantine (1 μM) prevented the Aβ-induced decrease of TrkB-FL receptors. Indeed, in these conditions, TrkB-FL increased (TrkB-FL_Aβ+Mem_: 0.99 ± 0.07; TrkB-FL_Aβ_: 0.67 ± 0.06, *n* = 9–10, *p* = 0.006, two-way ANOVA with Tukey *post hoc* test, **Figure 1A**) and TrkB-ICD decreased, relatively to exposure to Aβ_25–35_ alone (TrkB-ICD_Aβ+Mem_: 1.02 ± 0.09; TrkB-ICD_Aβ_: 1.53 ± 0.10, *n* = 10–11, *p* = 0.002, two-way ANOVA with Tukey *post hoc* test, **Figure 1B**), to levels similar to those of control conditions. Memantine alone had no significant effect on TrkB-FL (*n* = 9, *p* = 0.120, two-way ANOVA with Tukey *post hoc* test, **Figure 1A**) or TrkB-ICD expression levels (*n* = 12, *p* = 0.886, two-way ANOVA with Tukey *post hoc* test, **Figure 1B**).

Next, to evaluate if memantine could affect Aβ_25–35_-induced activation of calpains, αII-spectrin levels and the formation of calpain-specific spectrin breakdown products (SBDPs) were evaluated. αII-spectrin (250 kDa) is a major substrate for calpain and caspase-3 proteases, and its cleavage can produce breakdown products with distinct molecular weights. Specifically, calpains mediate the degradation of αII-spectrin to highly stable 150 kDa SBDPs (SBDP150). The presence of the calpain-cleaved fragments occurs early in neural cell pathology and may be indicative of necrotic and excitotoxic neuronal injury and death (Yan et al., 2012). Our data show that the exposure of neuronal cultures to Aβ_25–35_ resulted in high expression levels of SBDP150 (SBP150_Aβ_: 3.22 ± 0.40; SBP150_CTR_: 1.00 ± 0.24; *n* = 7–20, *p* < 0.001, two-way ANOVA with Tukey *post hoc* test, **Figure 1C** and Supplementary Table 3), strongly suggesting a marked activation of calpains as previously reported (Jerónimo-Santos et al., 2015). Importantly, memantine was able to prevent this effect, significantly reducing the levels of SBDP150 (SBP150_Aβ+Mem_:1.14 ± 0.37), when compared with Aβ_25–35_ alone (*n* = 7–8, *p* = 0.002, two-way ANOVA with Tukey *post hoc* test). Memantine (1 μM) alone had no significant effect upon calpain activity (*n* = 12–20, *p* = 0.994, two-way ANOVA with Tukey *post hoc* test **Figure 1C**).

N-methyl-d-aspartate receptors are tetramers of GluN1 and GluN2 subunits, with the latter being regarded as the subunit predominantly involved in excitotoxicity (Ferreira et al., 2012). To evaluate if the neuroprotective effects of memantine could correlate with changes in NMDAR subunit composition, we analyzed the levels of GluN2B and GluN1 subunits in primary neuronal cultured cells by WB. Interestingly, neither Aβ_25–35_ (25 μM) nor memantine (1 μM) significantly changed the expression levels of these subunits (Supplementary Tables 4, 5).

So far, our results suggest that, at least in our experimental conditions, memantine is able to prevent Aβ_25–35_-induced cleavage of TrkB-FL receptors, putatively by preferential blockade of eNMDAR (Xia et al., 2010) and through a mechanim independent of changes in NMDAR subunits expression levels.

### NMDAR Inhibition Restores the Ability of BDNF to Increase Spine Density of Cultured Cortical Neurons

Next, we aimed to further clarify the structural and functional implications, at the synaptic level, of memantine ability to prevent Aβ_25–35_-induced impairment of BDNF signaling.

Firstly, we evaluated spine density of cultured cortical neurons as a morphological readout, since BDNF is known to positively modulate the number of dendritic spines (Tyler and Pozzo-Miller, 2001; Ji et al., 2005, 2010; Kellner et al., 2014).

Our data showed that exposure to BDNF (20 ng/mL) alone, for 24 h, significantly increased spine density, compared with control conditions (BDNF: 8.55 ± 0.28 vs. CTR: 7.34 ± 0.25, *n* = 11–14, *p* = 0.044, three-way ANOVA with Tukey *post hoc* test, **Figure 3C** and Supplementary Table 6). Conversely, the number of dendrites quantified in cultured neurons exposed to Aβ_25–35_ (25 μM) for 24 h was significantly reduced (Aβ: 5.21 ± 0.27 vs. CTR: 7.34 ± 0.25, *n* = 12–14, *p* < 0.001, three-way ANOVA with Tukey *post hoc* test, **Figure 3C**). Moreover, BDNF (20 ng/mL) lost its effect when Aβ_25–35_ (25 μM) was simultaneously present (BDNF+Aβ: 7.45 ± 0.29 vs. BDNF: 8.55 ± 0.28, *n* = 11, *p* = 0.135, three-way ANOVA with Tukey *post hoc* test, **Figure 3C**). However, this loss of effect was prevented in the presence of memantine (1 μM) (BDNF+ Aβ+MEM: 9.03 ± 0.36 vs. BDNF+Aβ: 7.45 ± 0.29, *n* = 7–11, *p* = 0.021, three-way ANOVA with Tukey *post hoc* test, **Figure 3C**). Memantine (1 μM) *per se* was able to prevent Aβ_25–35_-induced decrease in spine density (Aβ+MEM: 6.41 ± 0.33 vs. Aβ: 5.21 ± 0.27, *n* = 8–12, *p* = 0.037, three-way ANOVA with Tukey *post hoc* test, **Figure 3C**). Interestingly, memantine alone decreased spine density (MEM: 4.75 ± 0.39 vs. CTR: 7.34 ± 0.25, *n* = 6–14, *p* < 0.001, three-way ANOVA with Tukey *post hoc* test, **Figure 3C**). This can probably be explained by the fact that, in the absence of a neuronal insult, NMDAR activation might be required for neuronal homeostasis (Hunt and Castillo, 2012).

Next, we evaluated the contribution of calpains to the Aβ-mediated BDNF signaling impairment, on the same morphological readout. To this end, we used calpastatin (1 μM), a calpains inhibitor. Similarly to the results with memantine, calpastatin prevented both Aβ_25–35_-induced decrease in spine density (Aβ+CAL: 7.90 ± 0.30 vs. Aβ: 5.21 ± 0.27, *n* = 6–12, *p* < 0.001, three-way ANOVA with Tukey *post hoc* test, **Figure 3D** and Supplementary Table 7), as well as BDNF loss of effect in cultured neurons also exposed to Aβ_25–35_ (BDNF+Aβ+CAL: 9.43 ± 0.28 vs. BDNF+Aβ: 7.45 ± 0.29, *n* = 9–11, *p* < 0.001, three-way ANOVA with Tukey *post hoc* test, **Figure 3D**). Consistently, calpastatin alone did not significantly affect the number of dendritic protrusions (CAL: 7.25 ± 0.35 vs. CTR: 7.34 ± 0.25, *n* = 6–14, *p* = 1.000, three-way ANOVA with Tukey *post hoc* test, **Figure 3D**).

Altogether, these results suggest that both NMDAR blockade by memantine and calpain inhibition by calpastatin were able to restore BDNF effect on dendritic growth and to prevent Aβ_25–35_ deleterious effect on spine density. Since we have shown that NMDAR activation is upstream of calpain activation (**Figure 1**), we argue that the NMDAR/Ca^2+^/calpains pathway is mechanistically involved in mediating Aβ_25–35_-driven BDNF signaling impairment, as evaluated by exogenous BDNF effect on dendritic protrusions of cultured neurons.

### The Inhibition of NMDAR Can Partially Rescue the Facilitatory Effect of BDNF Upon LTP

Given the possibility that NMDAR activation could be involved in TrkB-FL cleavage by calpains and consequently involved in BDNF loss of function, we next aimed to evaluate whether NMDAR blockade by memantine could also rescue the action of BDNF on LTP in hippocampal slices pre-exposed to Aβ (Jerónimo-Santos et al., 2015).

As previously shown (Fontinha et al., 2008), the 3×3 𝜃-burst paradigm delivered in the presence of BDNF (20 ng/mL) induced a robust LTP, which was significantly higher than that obtained in the absence of BDNF (LTP_BDNF_: 38.46 ± 10.46% vs. LTP_CTR_: 14.10 ± 3.50%, *n* = 4, *p* = 0.049 paired *t*-test, **Figure 4B**). However, when hippocampal slices were pretreated with Aβ_25–35_ (25 μM) for 3 h, BDNF (20 ng/mL) failed to significantly enhance LTP magnitude (LTP_Aβ_: 17.68 ± 11.25% vs. LTP_Aβ+BDNF_: 20.93 ± 7.95%, *n* = 3, *p* = 0.825, paired *t*-test, **Figure 4D**). When hippocampal slices were simultaneously pre-treated with Aβ_25–35_ (25 μM) and memantine (1 μM) for 3 h, the enhancement of LTP magnitude induced by BDNF was restored (LTP_Aβ+Mem+BDNF_: 31.77 ± 8.59% vs. LTP_Aβ+Mem_: 19.35 ± 4.14%, *n* = 7, *p* = 0.026, paired *t*-test, **Figure 4F**). Interestingly, although CA1 hippocampal 𝜃-burst LTP is known to be NMDAR-dependent (Citri and Malenka, 2008), pre-treatment with memantine did not significantly affect LTP, in experimental conditions similar to ours (Pinho et al., 2017).

Altogether, our data shows that memantine was able to rescue BDNF boosting effect upon LTP in slices exposed to Aβ_25–35_. Thus, our results strongly suggest that Aβ-mediated impairment of BDNF action relies, at least partially, on a NMDAR-depended mechanism, also for functional synaptic outcomes.

## Discussion

Taken together, our results suggest that Aβ_25–35_ induces TrkB-FL cleavage through calpains that are activated, at least partially, by Ca^2+^ influx through NMDARs, which likely occurs at extrasynaptic sites, given the pharmacodynamic properties of memantine. TrkB-FL cleavage culminates in BDNF signaling disruption, as highlighted by BDNF loss of function on dendritic growth and synaptic potentiation. Conversely, when NMDARs were inhibited by memantine, loss of BDNF signaling was prevented, with beneficial structural and functional implications, at the synaptic level.

Brain-derived neurotrophic factor is a neurotrophin with key regulatory actions, on neuronal survival (Middleton et al., 2000), structural remodeling of excitatory spine synapses (Alonso et al., 2004), as well as in dendritic growth (Kumar et al., 2005) and plasticity (Arevalo and Wu, 2006). It acts through the activation of TrkB-FL receptors, which are coupled to three different signaling pathways: (i) the phosphatidylinositol-3-kinase (PI3K)/Akt pathway, (ii) the Ras/MAPK pathway, and (iii) the PLCγ pathway. Working together, these pathways underlie important cognitive processes, including memory formation. Apart from its full-length cognate receptor (TrkB-FL), BDNF can also bind to TrkB-TC, whereby a negative feedback on TrkB-FL signaling is activated (Eide et al., 1996; Dorsey et al., 2006). Consequently, many neuronal excitotoxic conditions are associated with downregulation of TrkB-FL and upregulation of TrkB-TC expression (Gomes et al., 2012). Moreover, BDNF levels were reported to be decreased in the CNS and in the blood of AD patients, which might indicate that BDNF is involved in the pathogenesis of this disease (Song et al., 2015). Furthermore, changes in BDNF signaling can lead to synaptic dysfunction that can account for memory deficits observed in AD (Arancio and Chao, 2007; Schindowski et al., 2008).

We have previously demonstrated that Aβ-induced impairment of BDNF actions on hippocampal LTP and neurotransmitter release were rescued when calpains were inhibited (Jerónimo-Santos et al., 2015). Given the central role of BDNF in synaptic physiology, a complete mechanistic understanding of its loss of function is of the utmost importance. Since calpains are overactivated by increased levels of Ca^2+^ (Kelly and Ferreira, 2006, 2007), we hypothesized that NMDARs could be implicated in the calpain-mediated cleavage of TrkB-FL. Indeed, NMDARs are an important homeostatic source of Ca^2+^ influx, but are also known to be involved in pathophysiological processes. Memantine – 1-amino-3,5-dimethyladamantane – is a commercially available drug currently used in clinical practice for the treatment of AD patients (Rogawski and Wenk, 2003; McShane et al., 2006; Parsons et al., 2007). It is an uncompetitive NMDA receptor antagonist with strong voltage dependency and rapid unblocking kinetics (Chen et al., 1992; Parsons et al., 1993). In addition, memantine has been used in several studies to selectively block the pathological activation of NMDARs, while leaving their physiological functions intact (Chen et al., 1992; Leveille et al., 2008; Papadia et al., 2008; Pinho et al., 2017). Thus, in the present study, we used memantine as a pharmacological tool to better understand NMDAR involvement in the mechanisms underlying TrkB-FL cleavage and its consequences.

Remarkably, we found that the cleavage of TrkB-FL in cortical cultured neurons treated with Aβ_25–35_ was prevented in the presence of memantine. This suggests that Aβ-induced NMDAR activation may contribute to the increased intracellular Ca^2+^ levels responsible for calpain activation and, subsequently, for TrkB-FL truncation. This is consistent with previous work showing that Aβ oligomeric species evoke an immediate rise in intracellular Ca^2+^ through activation of GluN2B subunit (Rönicke et al., 2011; Ferreira et al., 2012). NMDAR activation was shown to be upstream of calpain activation (del Cerro et al., 1994). Consistently, blockade of GluN2B prevented disruption of Ca^2+^ homeostasis induced by Aβ (Ferreira et al., 2012).

It is important to note, however, that distinct isoforms of calpains are differentially activated by synaptic and extrasynaptic NMDARs. In fact, synaptic NMDARs prefentially activate μ-calpains, while m-calpains are activated by eNMDAR (Parsons and Raymond, 2014). We did not evaluate the independent activation of different sets of calpains. However, it was previously shown that m-calpains induce the proteolysis of striatal-enriched protein phosphatase (STEP), activating the p38 mitogen-activated protein kinase (p38MAPK), wich culminates in cell death (Xu et al., 2009; Wang et al., 2013). Thus, we hypothesize that the overactivated calpains that are in the pathway culminating in TrkB cleavage are, most likely, of the m-subtype.

Brain-derived neurotrophic factor is known to facilitate or to boost hebbian plasticity mechanisms, which, in turn, rely on the formation of dendritic spines (Tartaglia et al., 2001; Knobloch and Mansuy, 2008). Therefore, we also evaluated whether NMDAR inhibition by memantine could prevent the Aβ_25–35_-induced BDNF loss of function upon spine density and synaptic potentiation. The results show that inhibition of NMDARs or calpains by memantine or calpastatin, respectively, was capable to restore BDNF effects on spine density in cultured cortical neurons, previously exposed to Aβ_25–35_, thus suggesting that NMDAR activation, probably upstream of calpain overactivation, contributes to Aβ-mediated BDNF loss of function upon spine density. This is consistent with previous results demonstrating that BDNF has important roles in spine outgrowth (Tyler and Pozzo-Miller, 2001; Ji et al., 2005, 2010; Kellner et al., 2014), which are impaired by Aβ (Shrestha et al., 2006; Smith D.L. et al., 2009). In addition, alterations in spine density are thought to contribute to cognitive deficits (Knobloch and Mansuy, 2008). Furthermore, it has been shown that NMDAR inhibition prevents the decrease in synaptic density in several animal models of AD (Shankar et al., 2007; Wei et al., 2010), which is in agreement with the data herein reported on cortical cultured neurons.

However, whether such NMDAR excessive activation is associated with changes in NMDAR subunits expression is not yet clear. Some evidence suggests that GluN1 mRNA levels are unchanged in AD patient’s brains (Bi and Sze, 2002), which is consistent with the results we obtained. On the other hand, it has been reported that expression of GluN2B mRNA and protein levels are decreased in the hippocampus and cortex of *post-mortem* human AD brain (Bi and Sze, 2002; Hynd et al., 2004; Mishizen-Eberz et al., 2004). It is possible that certain subunit combinations may be lost in AD because of selective neuronal loss. If so, retention of GluN1 transcripts suggests that neurons that express GluN2B NMDAR are comparatively more susceptible to neurotoxicity (Hynd et al., 2004). Other reports, however, showed that Aβ oligomers induced an increase of GluN2B membrane expression at extrasynaptic sites (Gilson et al., 2015). We did not observe significant changes in GluN2B protein levels in our experimental conditions. These discrepancies may be related with differences between AD models; namely, in our experimental setup, neurons were acutely exposed to Aβ_25–35_, which does not recapitulate the chronic and slow build-up of Aβ in the brains of AD patients. It may be that, in the early stages of AD before symptomatic onset, Aβ modulates the activity of NMDARs, putatively acting as a trigger to neurodegeneration. Furthermore, Aβ is known to enhance GluN2B-mediated NMDA currents and extrasynaptic responses. Thus, changes in NMDAR activity rather than changes in NMDAR composition may be early involved in the neurodegenerative cascade.

Long-term potentiation, widely accepted as the molecular substrate for learning and memory (Bliss and Collingridge, 1993), was used as a functional readout to evaluate the consequences of NMDAR inhibition. BDNF has the capacity to increase LTP magnitude through TrkB-FL activation (Korte et al., 1995; Figurov et al., 1996; Minichiello et al., 2002). Furthermore, LTP is correlated with the formation of new spines within minutes of induction (Toni et al., 1999; Lüscher and Malenka, 2012). We previously demonstrated that Aβ-induced impairments of BDNF actions upon hippocampal LTP are dependent on calpain activation (Jerónimo-Santos et al., 2015). The data herein presented expands our understanding of these processes, since NMDARs inhibition by memantine was also able to restore BDNF effect upon LTP. Hence, we hypothesize that, also for LTP, eNMDAR activation should be upstream of calpain overactivation leading to BDNF signaling impairment.

It is important to highlight that Aβ_25–35_ *per se* decreases spine density on primary neuronal cultures but has no significant effect upon LTP on hippocampal slices. Previous reports have shown that Aβ_1–42_, Aβ_1–40_ and the active fragment Aβ_25–35_ can significantly impair LTP in rat hippocampal slices (Chen et al., 2000). However, other reports have shown no significant changes on LTP magnitude (Smith J.P. et al., 2009; Jerónimo-Santos et al., 2015). The developmental age and genetic background of the animals used, the stimulation protocol or Aβ preparation (Smith J.P. et al., 2009) could explain such absence of Aβ effect upon LTP.

N-methyl-d-aspartate receptors, which are composed by four subunits (Paoletti et al., 2013), can be subdivided in synaptic and extrasynaptic subtypes (Hardingham et al., 2002), depending on their subcellular localization. NMDAR containing GluN2B are predominantly located extrasynaptically, while GluN2A-containing receptors have been mainly found at synaptic domains (Cull-Candy et al., 2001). The concentration of memantine used in this study, 1 μM, is within a concentration range that is known to target extrasynaptic receptors selectively (Xia et al., 2010). Therefore, we advance the hypothesis that NMDARs located at extrasynaptic sites are preferentially responsible for the rise in intracellular Ca^2+^ induced by Aβ, leading to TrkB-FL cleavage and the consequent BDNF loss of function. In line with this thesis, eNMDARs are known to be excessively activated in the presence of Aβ and in animal models of AD (Li et al., 2011; Talantova et al., 2013; Hanson et al., 2015). Consistently, inhibition of GluN2B-NMDARs was shown to prevent or reverse some of the synaptic deficits in animal models (Paoletti et al., 2013; Zhou and Sheng, 2013). If BDNF signaling disruption is prevented in the presence of memantine and given that BDNF is thought to play important neuroprotective effects, it is intriguing that the clinical benefit of memantine is only moderate, at best (Folch et al., 2017). The present work was not designed to answer this question. However, given that Aβ build-up is known to begin many years before symptomatic presentation, we argue that, at the time patients are commonly started on memantine, BDNF signaling will already be severely compromised, with little margin for such prophylactic effect (Caselli and Reiman, 2013).

**FIGURE 5:**
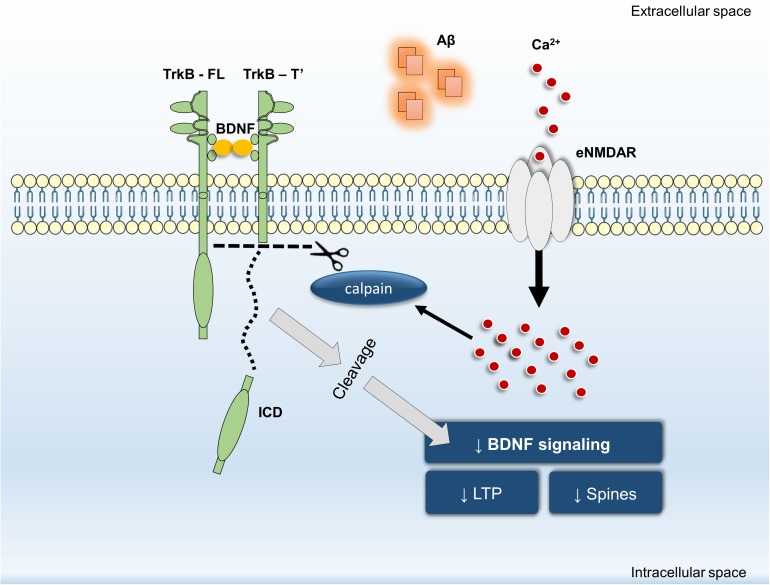
General overview of the main highlights of this work.

In summary, our data revealed that inhibition of Aβ_25–35_-induced NMDAR activation by memantine: (i) prevents TrkB-FL truncation and (ii) prevents BDNF loss of effect on structural (spine density) and functional outcomes (LTP magnitude). Furthermore, we have shown that calpain activation is downstream of NMDARs and that calpain inhibition prevented Aβ_25–35_-induced BDNF loss of effect on dendritic protrusions. Thus, we propose that NMDAR activation, particularly at extrasynaptic sites, is mechanistically involved in Aβ-triggered intracellular Ca^2+^ build-up, consequently leading to calpain activation, TrkB-FL receptor cleavage and BDNF signaling impairment (**Figure 5**). By detailing the mechanisms involved in Aβ-induced cleavage of TrkB receptors and in the early functional consequences of this dysregulation, these findings highlight the role of eNMDAR activation upon BDNF signaling dysregulation in excitotoxic conditions leading to the neuronal dysfunction occurring in AD.

## Ethics Statement

This study was carried out in accordance with the recommendations of “Directive 2010/63/EU.” The protocol was approved by the “iMM’s Institutional Animal Welfare Body – ORBEA-iMM and the National Competent Authority – DGAV (Direção-Geral de Alimentação e Veterinária).”

## Author Contributions

ST and RR contributed equally to this work and were responsible for study concept and design, performed the experiments, interpreted the results, and wrote the initial draft of the manuscript. TR analyzed the data and revised the manusript. LL and AS contributed to interpretation of results and revised the manuscript. MD was responsible for the concept and design of the study, supervised the work, contributed to interpretation of the results and revised the manuscript.

## Conflict of Interest Statement

The authors declare that the research was conducted in the absence of any commercial or financial relationships that could be construed as a potential conflict of interest.

## Abbreviations

Aβ: Amyloid-β peptide, aCSF, Artificial cerebrospinal fluid
AD: Alzheimer’s disease
BDNF: Brain-derived neurotrophic factor
eNMDAR: extrasynaptic N-methyl-d-aspartate receptor
fEPSPs: Field excitatory post-synaptic potential
HBSS: Hanks’ balanced salt solution
ICD: intracellular domain fragment
LTP: long-term potentiation
NMDAR: N-methyl-d-aspartate receptor
PBS: Phosphate-buffered saline
RT: Room temperature
SDS: Sodium dodecyl sulfate
SEM: Standard error of the mean
TrkB-FL: TrkB-full length
TrkB-T’: new truncated TrkB
TrkB-TC: truncated TrkB
