# Supplementary material for: Inhibition of NMDA Receptors Prevents the Loss of BDNF Function Induced by Amyloid β
Source: Front Pharmacol. 2018 Apr 11;9:237. doi: 10.3389/fphar.2018.00237 (PMC5904251; doi:10.3389/fphar.2018.00237)
Supplement: Supplementary file 1 [file Table_1.DOCX]

Supplementary Material

Inhibition of NMDA receptors prevents the loss of BDNF function induced by amyloid β

Sara Ramalho Tanqueiro, Rita Mira Ramalho, Tiago M. Rodrigues, Luísa V. Lopes, Ana Maria Sebastião, Maria José Diógenes*

*** Correspondence:** Maria José Diógenes, [diogenes@medicina.ulisboa.pt](mailto:diogenes@medicina.ulisboa.pt)

| **Supplementary Table 1.** Two-way ANOVA model for the effect of Aβ and memantine on TrkB-FL levels (relates to Figure 1A in the main text). MS. Mean Squares. | | | |
| --- | --- | --- | --- |
| Source | MS | *F* | *p* |
| Model | 0.303 | 7.07 | 0.0005 |
| Aβ | 0.063 | 1.46 | 0.2330 |
| Memantine | 0.055 | 1.28 | 0.2645 |
| Aβ x Memantine | 0.718 | 16.75 | 0.0002 |

| Residual | 0.043 |  |  |
| --- | --- | --- | --- |
