# Supplementary material for: Inhibition of NMDA Receptors Prevents the Loss of BDNF Function Induced by Amyloid β
Source: Front Pharmacol. 2018 Apr 11;9:237. doi: 10.3389/fphar.2018.00237 (PMC5904251; doi:10.3389/fphar.2018.00237)
Supplement: Supplementary file 2 [file Table_2.DOCX]

Supplementary Material

Inhibition of NMDA receptors prevents the loss of BDNF function induced by amyloid β

Sara Ramalho Tanqueiro, Rita Mira Ramalho, Tiago M. Rodrigues, Luísa V. Lopes, Ana Maria Sebastião, Maria José Diógenes*

*** Correspondence:** Maria José Diógenes, [diogenes@medicina.ulisboa.pt](mailto:diogenes@medicina.ulisboa.pt)

| **Supplementary Table 2.** Two-way ANOVA model for the effect of Aβ and memantine on TrkB-ICD levels (relates to Figure 1B in the main text). MS. Mean Squares. | | | |
| --- | --- | --- | --- |
| Source | MS | *F* | *p* |
| Model | 0.705 | 7.57 | 0.0003 |
| Aβ | 0.694 | 7.46 | 0.0088 |
| Memantine | 0.550 | 5.91 | 0.0188 |
| Aβ x Memantine | 1.090 | 11.71 | 0.0013 |
| Residual | 0.093 |  |  |
