# Supplementary material for: Inhibition of NMDA Receptors Prevents the Loss of BDNF Function Induced by Amyloid β
Source: Front Pharmacol. 2018 Apr 11;9:237. doi: 10.3389/fphar.2018.00237 (PMC5904251; doi:10.3389/fphar.2018.00237)
Supplement: Supplementary file 3 [file Table_3.DOCX]

Supplementary Material

Inhibition of NMDA receptors prevents the loss of BDNF function induced by amyloid β

Sara Ramalho Tanqueiro, Rita Mira Ramalho, Tiago M. Rodrigues, Luísa V. Lopes, Ana Maria Sebastião, Maria José Diógenes*

*** Correspondence:** Maria José Diógenes, [diogenes@medicina.ulisboa.pt](mailto:diogenes@medicina.ulisboa.pt)

| **Supplementary Table 3.** Two-way ANOVA model for the effect of Aβ and memantine on SBDP150 levels (relates to Figure 1C in the main text). MS. Mean Squares. | | | |
| --- | --- | --- | --- |
| Source | MS | *F* | *p* |
| Model | 9.921 | 8.97 | 0.0001 |
| Aβ | 15.171 | 13.72 | 0.0006 |
| Memantine | 11.849 | 10.71 | 0.0021 |
| Aβ x Memantine | 9.758 | 8.82 | 0.0048 |
| Residual | 1.106 |  |  |
