# Supplementary material for: Inhibition of NMDA Receptors Prevents the Loss of BDNF Function Induced by Amyloid β
Source: Front Pharmacol. 2018 Apr 11;9:237. doi: 10.3389/fphar.2018.00237 (PMC5904251; doi:10.3389/fphar.2018.00237)
Supplement: Supplementary file 5 [file Table_5.DOCX]

Supplementary Material

Inhibition of NMDA receptors prevents the loss of BDNF function induced by amyloid β

Sara Ramalho Tanqueiro, Rita Mira Ramalho, Tiago M. Rodrigues, Luísa V. Lopes, Ana Maria Sebastião, Maria José Diógenes*

*** Correspondence:** Maria José Diógenes, [diogenes@medicina.ulisboa.pt](mailto:diogenes@medicina.ulisboa.pt)

| **Supplementary Table 5.** Two-way ANOVA model for the effect of Aβ and memantine on GluN2B levels (relates to Figure 2B in the main text). MS. Mean Squares. | | | |
| --- | --- | --- | --- |
| Source | MS | *F* | *p* |
| Model | 0.095 | 1.18 | 0.3443 |
| Aβ | 0.063 | 0.79 | 0.3869 |
| Memantine | 0.115 | 1.44 | 0.2456 |
| Aβ x Memantine | 0.119 | 1.49 | 0.2384 |
| Residual | 0.080 |  |  |
