# Supplementary material for: Inhibition of NMDA Receptors Prevents the Loss of BDNF Function Induced by Amyloid β
Source: Front Pharmacol. 2018 Apr 11;9:237. doi: 10.3389/fphar.2018.00237 (PMC5904251; doi:10.3389/fphar.2018.00237)
Supplement: Supplementary file 6 [file Table_6.DOCX]

Supplementary Material

Inhibition of NMDA receptors prevents the loss of BDNF function induced by amyloid β

Sara Ramalho Tanqueiro, Rita Mira Ramalho, Tiago M. Rodrigues, Luísa V. Lopes, Ana Maria Sebastião, Maria José Diógenes*

*** Correspondence:** Maria José Diógenes, [diogenes@medicina.ulisboa.pt](mailto:diogenes@medicina.ulisboa.pt)

| **Supplementary Table 6.** Three-way ANOVA model for the effect of Aβ, memantine and BDNF on the number of dendritic spines (relates to Figure 3C in the main text). MS. Mean Squares. | | | |
| --- | --- | --- | --- |
| Source | MS | *F* | *p* |
| Model | 21.527 | 23.75 | <0.0001 |
| Aβ | 0.307 | 0.34 | 0.5630 |
| Memantine | 0.307 | 0.34 | 0.5624 |
| BDNF | 50.420 | 55.62 | <0.0001 |
| Aβ x Memantine | 32.077 | 35.38 | <0.0001 |
| Aβ x BDNF | 3.907 | 4.31 | 0.0420 |
| Memantine x BDNF | 0.324 | 0.36 | 0.5520 |
| Residual | 0.907 |  |  |
