# Supplementary material for: Inhibition of NMDA Receptors Prevents the Loss of BDNF Function Induced by Amyloid β
Source: Front Pharmacol. 2018 Apr 11;9:237. doi: 10.3389/fphar.2018.00237 (PMC5904251; doi:10.3389/fphar.2018.00237)
Supplement: Supplementary file 7 [file Table_7.DOCX]

Supplementary Material

Inhibition of NMDA receptors prevents the loss of BDNF function induced by amyloid β

Sara Ramalho Tanqueiro, Rita Mira Ramalho, Tiago M. Rodrigues, Luísa V. Lopes, Ana Maria Sebastião, Maria José Diógenes*

*** Correspondence:** Maria José Diógenes, [diogenes@medicina.ulisboa.pt](mailto:diogenes@medicina.ulisboa.pt)

| **Supplementary Table 7.** Three-way ANOVA model for the effect of Aβ, calpastatin and BDNF on the number of dendritic spines (relates to Figure 3D in the main text). MS. Mean Squares. | | | |
| --- | --- | --- | --- |
| Source | MS | *F* | *p* |
| Model | 18.521 | 25.36 | <0.0001 |
| Aβ | 4.572 | 6.26 | 0.0149 |
| Calpastatin | 21.313 | 29.18 | <0.0001 |
| BDNF | 34.225 | 46.86 | <0.0001 |
| Aβ x Calpastatin | 17.222 | 23.58 | <0.0001 |
| Aβ x BDNF | 3.020 | 4.13 | 0.0462 |
| Calpastatin x BDNF | 1.214 | 1.66 | 0.2020 |
| Residual | 0.730 |  |  |
